# Citizens’ economic recovery models for a pandemic

**DOI:** 10.1371/journal.pone.0266531

**Published:** 2023-02-03

**Authors:** Asmus Leth Olsen, Anders Woller

**Affiliations:** Dept Political Science, University of Copenhagen, Copenhagen, Denmark; University of Edinburgh, UNITED KINGDOM

## Abstract

The COVID-19 pandemic brought sudden economic devastation and forced countries to respond with policies to counter the looming economic crisis. What policy response do citizens prefer to combat an economic decline due to a pandemic? We study the preferences of citizens regarding economic policy and changes in these preferences as the pandemic unfolded in Denmark. Denmark passed early and comprehensive legislation with broad support from all political parties to counter the economic crisis caused by the pandemic. We employ a large nationally representative two-wave panel of Danish citizens (N = 12,131) drawn from the administrative registers, from which data was collected at the onset of the economic shock and immediately prior to economic recovery. In both waves the same subjects describe their preferred economic solution to COVID-19 in open-text format. We generate a simple dictionary method to uncover a set of distinct laymen economic policy responses. First, we find that citizens formulated a diverse set of policy interventions. Second, citizens become markedly stronger proponents of economic intervention as the crisis unfolded. Finally, we show how differences in economic preferences across partisanship vanished during the crisis.

## Introduction

Like a whiplash, the COVID-19 pandemic wreaked havoc across the world’s economies. As a consequence of this unprecedented shock, policy makers have been forced to consider measures to save the economy from complete collapse [1]. Since the financial crisis in 2008, there has been a revival of interest in possible policy interventions to combat economic crises [2–4] and the political consequences of different government responses [5, 6]. In democracies, citizen preferences and beliefs, at least ideally, determine and constrain what initiatives are politically feasible—including economic crisis responses to the pandemic [7, 8]. This study maps citizens’ attitudes to handling the economy and contributes to previous studies on the attitudes surrounding the management of the health system in response to the pandemic [9, 10]. The wide palette of economic policy initiatives and the constraint of the electorate raises the fundamental question of what preferences citizens hold regarding the economic recovery from a pandemic like COVID-19? We approach this question with a rich nationally representative two-wave panel survey of Danish citizens (N = 12,131) drawn from the Danish administrative records. Featured in the *New York Times* in late March 2020 under the headline “The Nordic Way to Economic Rescue” [11], the Danish case is particularly interesting as the economic response was swift, comprehensive, supported by all political parties, and largely successful [12, 13]. Our study explores the underlying structure of preferences among Danes that allowed for this style of economic policy intervention.

In our study, subjects were interviewed twice, with the first wave of interviews fielded in early April 2020. For the second wave, the same subjects received a re-invitation on a randomized date between mid-June and end of July. To measure subjects’ economic preferences in each wave, we prompted them to elaborate on their preferred economic policy solutions to COVID-19 in free form text. Using a dictionary method [14], we categorize the spontaneous policy preferences that citizens express about economic recovery following COVID-19. The panel structure, as well as the randomized re-invitations, uniquely situates us to investigate (1) the distribution of preferences over economic recovery in the population, (2) how an economic crisis affects the support for particular policies, and (3) if heterogeneity exists in terms of how individual-level partisan characteristics correlate with specific economic preferences over time.

Our analysis reveals that citizens are able to formulate a diverse set of economic policy interventions, with eight major distinct solutions each supported by more than five percent of subjects (Fig 3B). We find that the overall preference for a policy intervention increases markedly throughout the crisis. Whereas 42.5% suggest active intervention with economic policy in the first wave, 84.9% of the same subjects suggest the same in the second wave. Furthermore, an initial partisan divide over whether to intervene or not dwindles. Thus, in the first wave, 70% of left-leaning party voters suggest expansionary economic policy, compared to about 60% of right-leaning party voters. However, in the second wave, for the same individuals, the numbers are 91% and 88%, respectively. As a consequence of the unfolding of the crisis, only minor partisan differences remain with respect to government intervention; differences which are stable across the second wave study period. We also show how partisan differences re-emerge slightly as differences in preferences for various *types* of government intervention.

## Context

**Fig 1:**
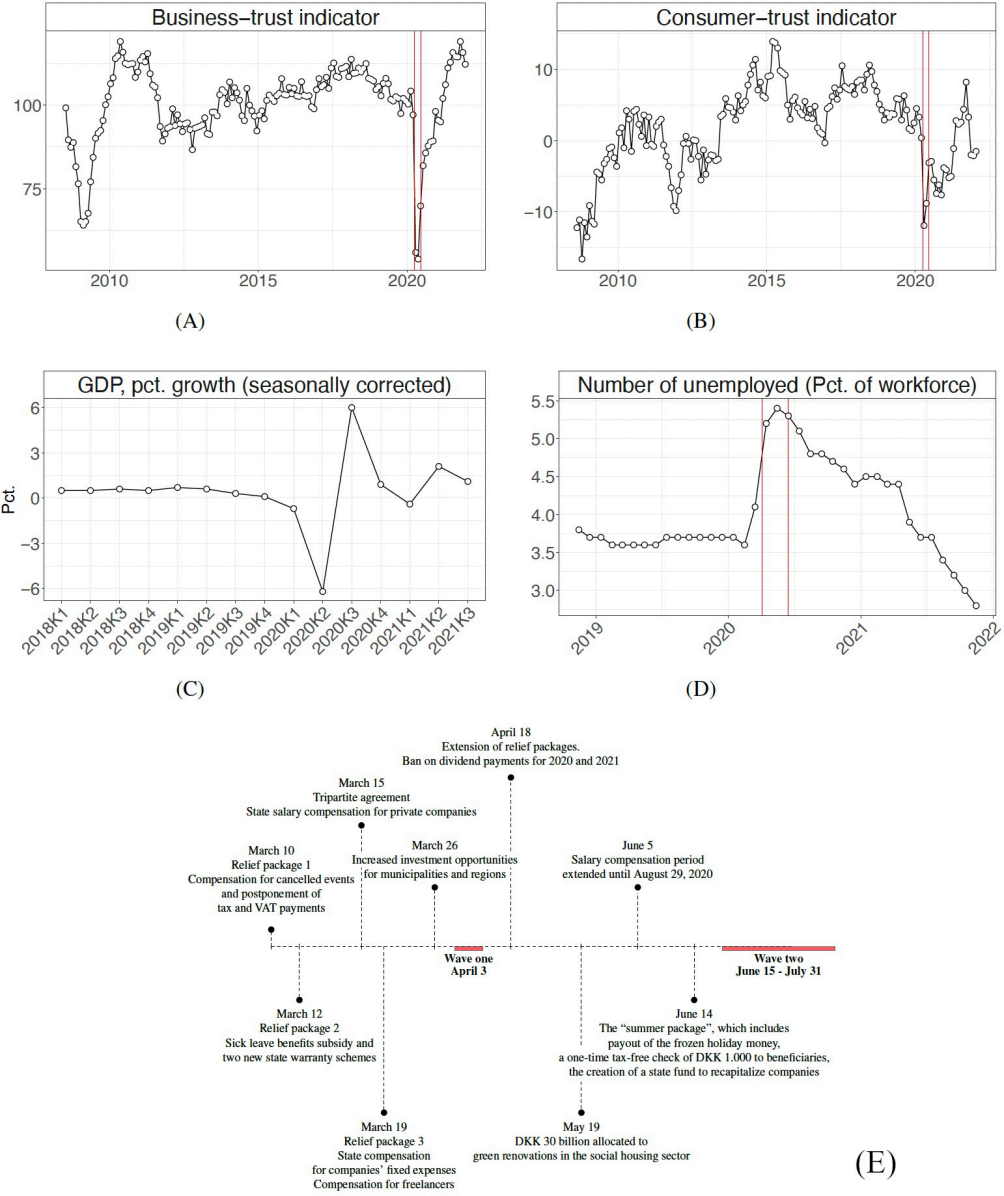
Development in business (Panel A, monthly) and consumer trust (Panel B, monthly), GDP (Panel C, quarterly) and unemployment (Panel D, monthly). Panel E shows economic initiatives to combat COVID-19. Red lines indicate invitation to the two survey waves.

Like the rest of the world, Denmark was hit by the COVID-19 pandemic [15], which resulted in an unprecedented decline in economic activity following the state-prescribed lock-down of all non-essential public institutions as well as businesses. According to the official statistics agency in Denmark, unemployment increased 22% in April and May [16] and between the first and the second quarter of 2020, the Danish GDP experienced negative growth of 7.4% [17]. The unprecedented hit to the economy happened right at the onset of our first wave of interviews, but before our second wave. The dramatic shock to the economy is illustrated in Fig 1. According to the business-, and consumer-trust indicators [18], the state of the economy in April and May was comparable to the worst period during the 2008 financial crisis. The hard hit to the economy is also illustrated in our survey: in the second wave, 10% of subjects report that their economic situation has suffered substantially from the pandemic.

In response to the severe economic decline, the Danish government implemented a range of economic support packages prior to our first wave of interviews, outlined in Panel C in Fig 1. These included compensation for cancelled arrangements, postponing payment deadlines, boosting the capacity of banks to lend money, and temporarily compensating businesses for their fixed expenses. See S1 Table for a full overview. Combined, the support packages totalled more than 200 Billion USD, or 34,500 USD per capita [19].

## Methods

We rely on a large nationally representative panel of Danish citizens that were interviewed twice during the COVID-19 pandemic. A random sample consisting of 100,000 adult Danes (18+ years, Danish citizens and permanent Danish address) was drawn from the Danish Civil Registration System (CPR) through The Danish Health Data Authority. The study complies with the Committee Act of the Danish National Committee of Health Research Ethics, which states that “Surveys using questionnaires and interviews that do not involve human biological material (section 14(2) of the Committee Act)” are exempted from approval (see https://en.nvk.dk/how-to-notify/what-to-notify). GDPR approval was provided by the Faculty of Social Sciences at the University of Copenhagen. The full sample was contacted using their personal government provided email account. Sampled individuals were sent reminders and a professional polling company telephoned subjects from under-represented groups in terms of gender, age, and region. We rely on responses from subjects that participated in both waves of the survey. This gives us a very large sample of 12,131 Danish citizens. The final sample is largely representative of the Danish population, but skewed slightly towards more highly educated and slightly older subjects (see S2 Table for descriptive statistics).

Two design features make us uniquely situated to explore the changes in laypeople’s economic beliefs following the economic shock illustrated in Fig 1. First, the survey is an individual panel survey: the first wave of surveys was fielded on April 3. Subjects that gave consent were re-invited for a second round of interviews beginning June 15 throughout July. On average, subjects answered the second wave 83 days after having responded to the wave one questions. This allows us to identify within-subject changes between the very early stages and after the initial economic shock.

Second, for our wave two survey, subjects were randomly sampled to receive the re-invitation at different points in time. Specifically, subjects were randomly distributed into daily batches of more than 600 subjects, and the batches covered each day over a 46-day period between June 15 and July 31. Due to the exogenous variation in time of response in the second wave, we are able to explore how changes in economic preferences persist over time. Compliance with the re-invitations was high: 45% percent of subjects replied within one day of the invitation, and 75% have replied within four days (see S1 Fig).

### Classifying open-ended answers

To measure subjects’ models of economic recovery, we make use of open-ended questions. Rather than defining the belief space ex-ante using predefined answer categories, open-ended answers allow us to exhaustively map the economic beliefs of subjects without priming them. Open-ended questions are well-suited to avoid demand effects and social desirability bias that would be inherent in any set of closed-ended items directly probing citizens about their preferences for various economic policy interventions [14], see e.g. [20, 21].

In the first wave, we asked the following question: “How do you think Denmark best avoids a prolonged economic crisis relating to the corona-pandemic?” Subjects were shown a text box immediately below the question where they could fill in their text answer. In the second wave, the setup was the same, but we changed the wording to further prompt answers that focused on economic policy. Here we asked: “The COVID-19 pandemic has hit the Danish economy hard. What is your best advice on which economic policy that is needed to get out of the crisis? We know that you are probably not an economist, but we would like to hear your opinion. There are no wrong answers.” Interpreting Fig 3 panel A the slight change in question wording does indeed seem to have reduced the number of answers having to do with health measures, which are irrelevant to the purpose of our study. In both surveys, the open text fields were preceded only by non-COVID and non-economics issues in order to avoid the risk of priming and educating subjects on feasible economic models. Subjects generally provided informative responses, with an average length of 27 words (see S2 and S3 Figs).

To categorize the open-ended answers with respect to economic preferences, we rely on a simple text-as-data approach known as a dictionary method. Specifically, we identify particular words or phrases related to particular categories of interest, and generate an original dictionary able to classify laypeople’s economic models. To generate the dictionary, we made use of a two-legged approach. First, we generated lists of words through close readings of 3,000 randomly drawn individual replies. Second, we relied on the media covering government responses, and derived particular words used to describe the range of economic solutions. The creation of the dictionary was characterized by an iterative process, where we paid particular attention to replies not yet captured by the dictionary. After having run through the process multiple times, only 11% of answers were not categorized.

S3 Table shows the number of words and match share for each category in the dictionary. S4 Table shows no statistically significant correlation between the number of words used to capture a category and its match share when excluding the three largest categories (demand side, classical, and health). To validate our measure, we use wordscore [22], a supervised scaling algorithm. We hand coded 100 reference categories with respect to intervention and non-intervention, respectively, and the algorithm scores the remaining replies. S5 Table finds a very strong correlation between the two approaches: a one standard deviation change in the wordscore variable corresponds to an increased likelihood of 18.4% to 28.6% that our dictionary measure categorizes a reply as favoring economic intervention. The strong correlation between the two very different approaches to measuring preferences for economic intervention suggests that we are in fact capturing an underlying dimension in the answers and that our coding is not simply an artefact of the specific approach to measuring preferences.

## Results

**Fig 2:**
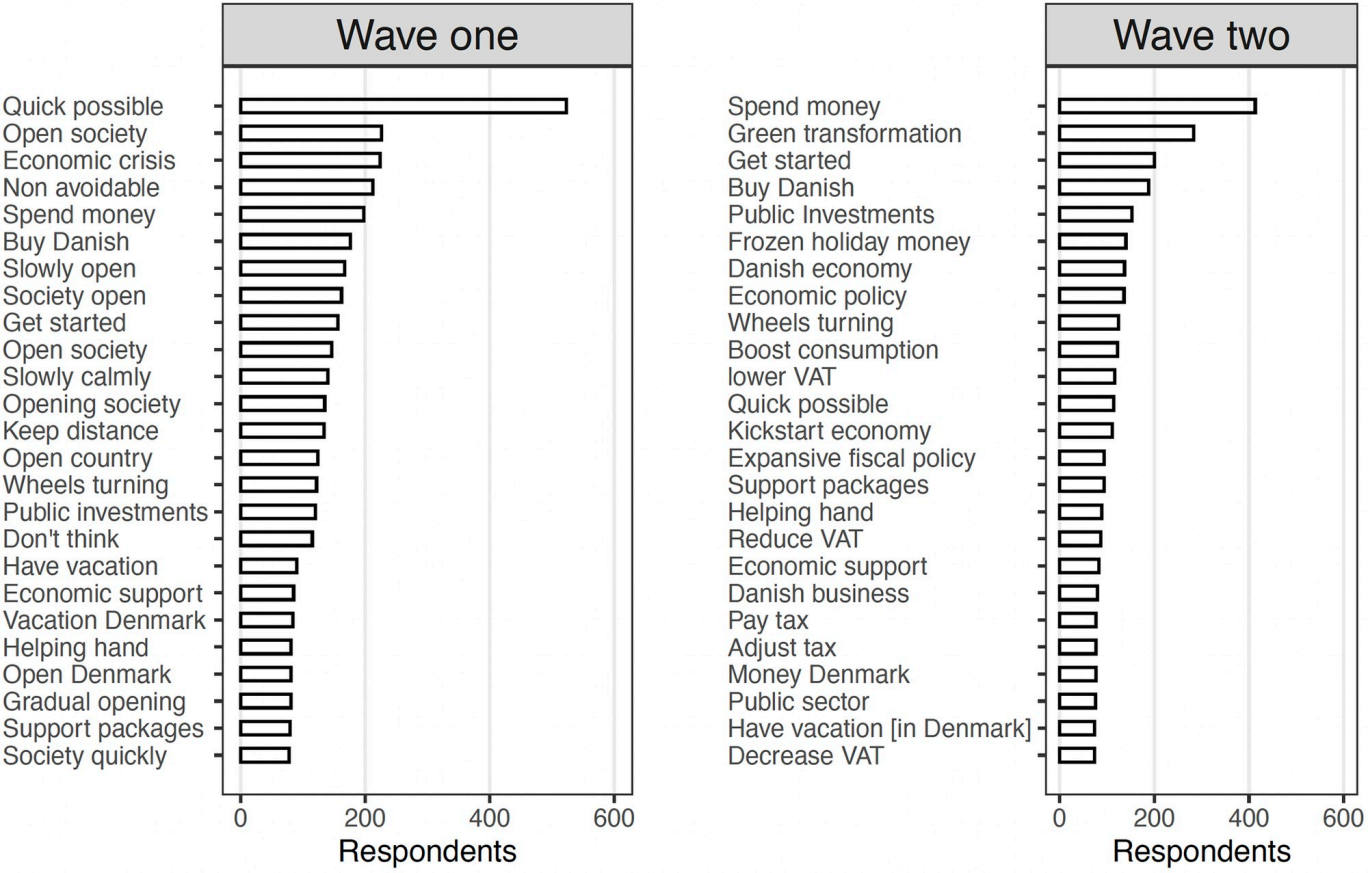
Most common bigrams in open-ended answers across the two waves.

Fig 2 shows the raw response data prior to any categorization. Specifically, it ranks the most common bigrams across the two waves. Several of the categories further explored below are visible simply from looking at the raw data. Of the most popular bigrams in the first wave, several relate to rolling back the state lock-down, including “Quick possible”, “Open society”, and “Slowly open”. In contrast, the most popular bigrams in the second wave seem to refer to state intervention, including “Spend money”, and “Green transformation”.

For both waves, but particularly the second wave, one can also see examples of suggestions for boosting the economy through spending money, public investments, and support packages. Some subjects furthermore specify that such investments should favor Danish businesses. Although asked specifically about economic solutions, some subjects focus on health measures (particularly wave one), including complying with the distance requirements set out by health authorities.

Overall, subjects seem to take one of two positions: that the state should intervene in the economy to boost demand, or the opposing view stressing simply that the state should roll-back the lock-down without the need for other measures. In our data, examples of the former include answers like “through active fiscal policy targeted core business”, “by maintaining employment, provide economic support to businesses, by securing money for continued consumption […]”, and “the market cannot solve this crisis, therefore the state needs to intervene with support packages”. In contrast, examples of the latter include “by reopening business again. Other than that, we shouldn’t continue sending support packages left and right […]”, “if we do not at least partly let the free market roam, we risk keeping some businesses artificially alive”, and “a well-thought out re-opening of the country in order to get the free market dynamic working”.

**Fig 3:**
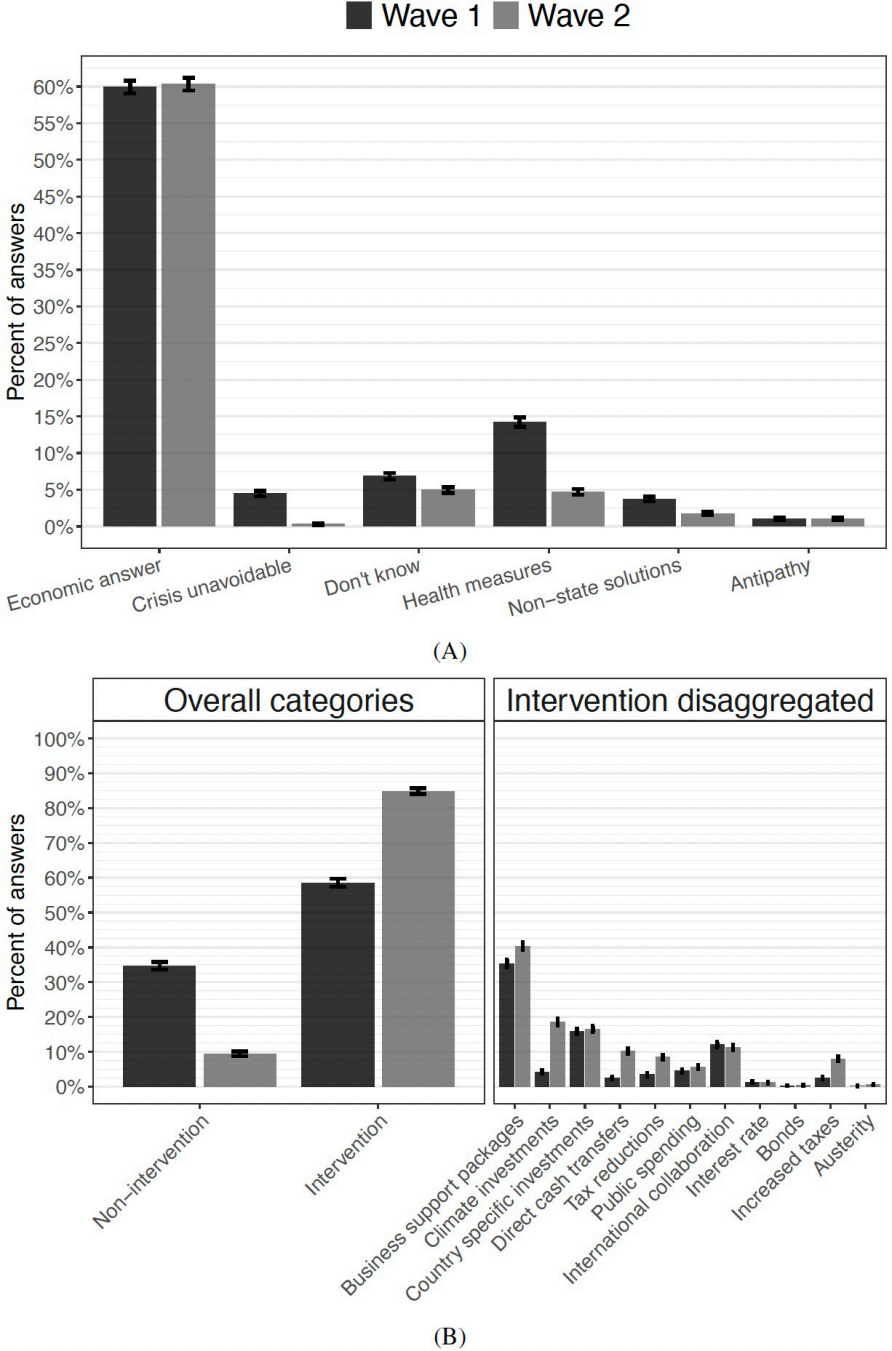
Panel A: Distribution across answer categories (all subjects). Panel B: Intervention vs. non-intervention and types of policy intervention (subjects providing economic answer). Lines are 95% confidence intervals.

Fig 3 panel A reveals aggregate differences in replies over time, specifically by comparing how the same group of subjects answered the question before and after the negative shock to the economy. The overall intervention category is created by aggregating all expansionary economic policy suggestions intended to boost demand into an overall intervention category. It includes increasing public spending, business support packages, climate/country specific investments, direct cash transfers, tax reductions, purchasing bonds, and/or lowered interest rates. Responses are categorized as intervention if any of these suggestions are identified. In contrast, non-intervention is identified when subjects utter a wish for no state intervention whatsoever and/or a preference for allowing the market forces to roam. Including only subjects who provided an *economic* answer in both rounds does not change results substantively (not shown). The share of subjects providing a substantive economic answer is close to constant at 60% across the two rounds. S4 Fig shows that older subjects with higher education are more likely to provide economic answers. Perhaps surprisingly, citizens also become less likely to believe that no economic solution exists. Citizens believing that an economic crisis is unavoidable has thus fallen from 4.2% to effectively zero.

Fig 3 panel B unpacks the economic solutions provided by subjects who did provide an economic answer. The left panel plots the two most aggregate economic categories identified in the data: non-intervention and intervention. Non-intervention is here defined as answers stating solely that the economy should be left to its own devises, e.g., by rolling back the lock-down, without stating any other necessary measures. Interestingly, the gap increases between the two survey rounds. Whereas the difference is 20.2 percentage points in wave one (22.3% compared to 42.5%), it increases to 75.5 percentage points in wave two (9.4% compared to 84.9%). It follows from the absolute increase in answers categorized as interventionist that intervention sub-categories increased as well. However, interpreting the right panel in Fig 3, some categories experienced a much larger increase in popularity than others. Although business support packages were already the most popular tool among subjects in the first round, the popularity increased by almost 15 percentage points to 40.3% of subjects in the second wave. In contrast, there was not widespread support for climate initiatives at the onset, but 18.5% supports such initiatives in the second wave—a five-fold increase. Direct cash transfers also increased five-fold, reaching 10% support in the second wave, likely as a result of increased political focus prior to the second wave of surveys (see S1 Table).

### Heterogeneity

**Fig 4:**
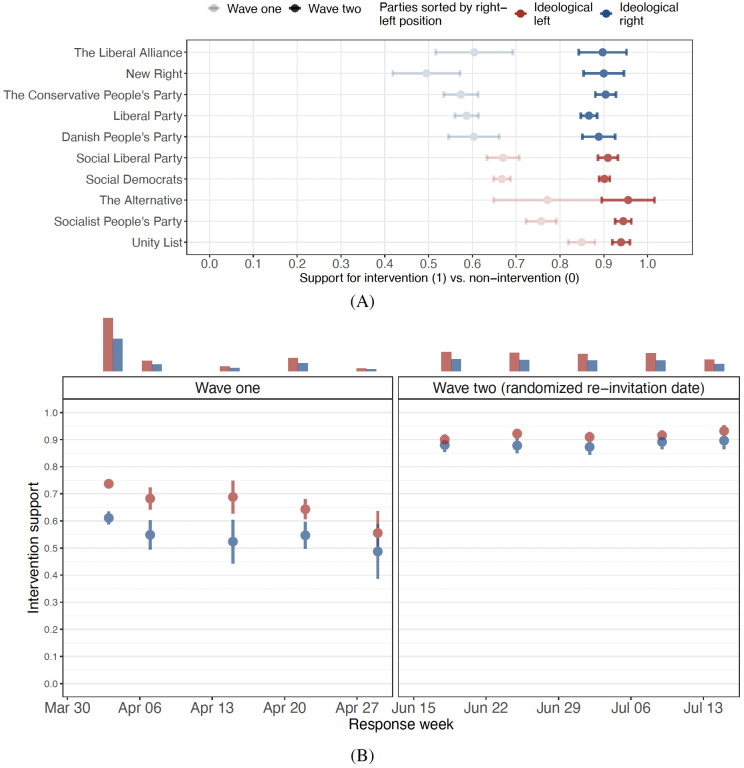
Panel A: Partisan responses to the COVID-19. Panel B: Differences before and after the negative economic shock. Red dots denote estimate means for center-left party voters and blue dots center-right party voters. Lines are 95% confidence intervals and bars reflect group sizes. Parties are sorted by their voters’ stance on economic inequality from necessary (top) to undesirable (bottom) from the Danish Election Survey [23].

We also captured subjects’ vote intention and ideological self placement by asking “In politics there is often talk about left and right. Where would you place yourself on this scale?” with eleven categories ranging from 0 (left) to 10 (right). Parties for which the average voter scores below 5 are categorized as “ideological left”. Initially in Wave 1, we find stark ideological differences in terms of whether to intervene in the economy. Specifically, we are able to predict the expected position of parties on a scale going from intervention to non-intervention with citizens with right-leaning party preferences being the more reluctant to intervene (see Panel A, Fig 4). For education, gender, and age there are no substantive difference in intervention preferences (see S5 Fig).

Interestingly, the economic shock erodes differences for the same individuals in wave two. Here, we no longer find any substantive or significant partisan divide over intervention: there is now agreement between left and right voters with respect to intervention. Furthermore, it is at a higher level for both groups than in wave 1. S6 Fig and S6 Table model the influence of party choice and ideological leaning on support for intervention, while introducing controls for age, gender, employment status, education, and fixed effects at the municipality and zip code, respectively. The insights from Fig 4 are supported when accounting for these background characteristics.

**Fig 5:**
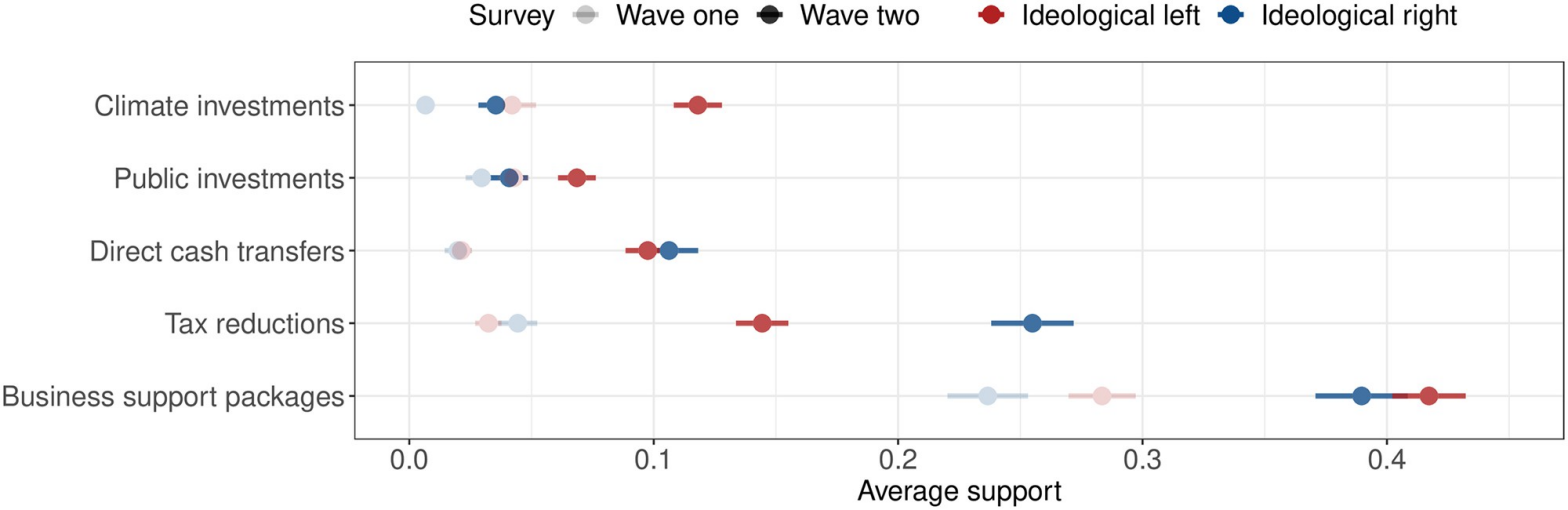
Support for different state interventions across ideology. Lines are 95% confidence intervals.

The randomized re-invitations to wave 2 allow us to track any re-emergence of partisan differences in the specific preferred intervention. Yet, over the months of June and July, we observe great stability in the convergence of and support for some kind of intervention in the economy (see Panel B, Fig 4). In fact, for the entire second wave, intervention support is within 90% ± 3 percentage points. This suggests that the impact of the crisis on the convergence in citizen preferences are persistent, at least in the period of study. The question arises: Does a partisan divide move from intervention vs. non-intervention to the specific type and form of intervention preferred. In order to study this, we look at the partisan split over the five most popular types of interventions as found in both wave 1 and wave 2 in Fig 5.

Fig 5 suggests that some partisan divides exist for different types of intervention. Whereas right-leaning voters are stronger proponents of tax reductions (25.4% compared to 14.5%), left-leaning voters support climate investments (11.9% compared to only 3.5%). Thus, the difference in support across ideological standpoints in the second wave is 11 percentage points, compared to only about 1 percentage point in the first wave. The same trend, although less pronounced, is visible for climate change and public investments. Results may suggest that large economic support packages to combat crises are politically feasibly, albeit politicians may have to show initial leadership.

## Conclusion

The COVID-19 pandemic has focused urgency on understanding how citizens think about economic policy solutions to a fast-moving and sudden economic shock. Our study was motivated by the simple fact that citizens’ preferences and expectations about economic recovery constrain and enhance certain policy tools that are available to policy makers under COVID-19: Citizens’ core preferences about recovery will be the ultimate basis for economic policy in mature democracies. Using open-ended responses from a large representative panel of Danes (N = 12,131), we have estimated the distribution and development of citizens’ preferences regarding economic recovery models in response to COVID-19 in a country that quickly passed a comprehensive economic crisis policy. In the initial weeks of COVID-19, citizens formulate both interventionist (42.5%) and non-interventionist (22.3%) responses to the crisis. Frequently mentioned interventionist policies include business support packages, climate investments, cash transfers, and tax reductions. As a consequences of the negative shock to the Danish economy, intervention preferences increased to 84.9%, with just 10% expressing non-interventionist beliefs. Furthermore, a moderate partisan divide over intervention in any shape or form that was clear at the onset of the crisis was no longer detectable: across left- and right-leaning positions, approximately 90% of voters commenting on the economy, supports intervention. Our results are in line with survey experimental evidence on citizens’ response to COVID-19. Studies have looked at citizens’ fiscal adjustment preferences and find that the partisan policy polarization largely disappears when citizens are exposed to information on predicted COVID-19 deaths and income losses [24]. Other studies find that partisanship does not modify the negative effect of being supplied with information on the consequences of COVID-19 on support for the incumbent president Donald Trump. Instead, the information supply reduces support across partisanship [25].

Our results are also align with experimental work showing that low polarized settings, such as the Danish political elite response to COVID-19, might foster greater voter emphasis on substantive information [26]; in our case, the serious economic situation caused by COVID-19. Our data on economic preferences tells a story of initial mild partisan polarization that eventually disappears as citizens of all partisan stripes converge on a preference for intervention in response to the crisis unfolding but with moderate partisan differences when discussing the *type* of intervention supported. Unsurprisingly, whereas right-leaning subjects tend to favor tax reductions to boost the economy, left-leaning voters are more likely to suggest climate investments. While our study is largely explorative, we can provide a tentative theoretical explanation of the observed development: Learning among voters from elites may have been affected by “partisan perceptional screen” [27], which introduces heterogeneity in learning across partisanship. Here, partisanship functions as a filter for information, causing adherents of different parties to perceive (economic) information differently from the same set of facts [28]. It can also affect how voters seek out information that is consistent with their prior ideological beliefs and dismiss or avoid information that is inconsistent with existing beliefs. Accordingly, voter polarization is often the product of elite polarization as voters learn about their favored party’s position on a policy issue [28]. Usually, the development of voter polarization follows the logic by which partisan predispositions are activated in the minds of citizens in response to elites debating an issue, which subsequently constrains the policy preferences of citizens [29]. For the case of economic recovery in Denmark, center-left voters were ideologically more inclined to believe in state intervention. At the same time, the ruling government—which was primarily responsible for the intervention—is center-left. Both aspects make center-left voters more inclined to support intervention from the onset. In contrast, center-right voters reason that any intervention impulse is to be avoided given that the opposing party block is in power. While this was true initially, center-right parties turned out to largely support intervention. That is, they did not send any diverging cues. As a consequence, center-right voters updated their intervention preferences and converged with center-left voters for a high level of agreement regarding the need for some form of economic intervention. Interestingly, previous studies have found that substantial shocks to the economy can speed up this convergence [30]. Under such circumstances, partisan rationalization may move elsewhere. This is consistent with our finding that the strong, negative shock to the economy erodes differences with respect to intervention versus non-intervention, instead moving the left/right disagreement to focus on the particular *type* of intervention as emphasized by individual parties in the negotiations.

## Supporting information

S1 Table Overview of economic policy to combat economic recession. (PDF)

S2 Table Subject covariates compared to the total population. (PDF)

S3 Table Descriptive data on dictionary categories. (PDF)

S4 Table Dictionary category words and match share. (PDF)

S5 Table Validation of dictionary measure with wordscore. Dictionary measure is a dummy taking the value 1 if a word indicating intervention is identified in the text. Wordscore is a supervised scaling algorithm [22]) that scores the individual answer by comparing it to a set of reference texts, which we code manually. Specifically, we manually code 100 answers with respect to intervention (1) or non-intervention (0). These texts act as anchor for the algorithm to score the remaining texts on a scale ranging from non-intervention to intervention. The association is estimated for four samples: the full sample and three samples excluding observations with the most extreme values on the wordscore measure. This is to prove that the association is not simply driven by clear and obvious (extreme) examples. Interpreting the table, we find a positive and statistically significant association between our dictionary and the wordscore measure. In other words, the two measures of economic intervention are in agreement. The association is furthermore practically important. Moving one standard deviation toward intervention on the wordscore measure corresponds to our dictionary measure being 3pct. to 13pct more likely to identify intervention in the same replies.
(PDF)

S6 Table Reduction in ideological polarization after the economic shock. Ideological right includes Danish Peoples’ Party, Liberals, The Conservative Peoples’ Party, The New Right, and Liberal Alliance. Ideological left is the remaining parties from Fig 4. Unemployed takes the form of a dummy. Education is a numeric variable indicating the highest education obtained (0 = primary school, 8 = PhD).
(PDF)

S1 Fig Compliance with re-invitations. (PDF)

S2 Fig Answer length across categories. (PDF)

S3 Fig Answer length across time. (PDF)

S4 Fig Providing economic answer across subject characteristics. (PDF)

S5 Fig Changes in support over time across background characteristics. (PDF)

S6 Fig Subject support for intervention across parties and surveys. Party positions relative to the Social Democrats (governing party). Models control for age, gender, employment status, and education and include fixed effects.
(PDF)
