# Supplementary material for: Citizens’ economic recovery models for a pandemic
Source: PLoS One. 2023 Feb 3;18(2):e0266531. doi: 10.1371/journal.pone.0266531 (PMC9897534; doi:10.1371/journal.pone.0266531)
Supplement: S1 Table — (PDF) [file pone.0266531.s001.pdf]

Variable	Category	Sample	Population
**Gender**			
(share)	Women	0.496	0.508
	Men	0.504	0.492
**Age**		55.5	50.5
(mean)			
**Education**			
(share)	Public school	0.062	0.101
	Vocational	0.212	0.383
	High school	0.077	0.117
	Further education (short)	0.122	0.059
	Further education (medium)	0.327	0.213
	Further education (long)	0.200	0.118
	PhD	0.017	0.009
**Region**			
(share)	Capital region	0.325	0.330
	Zealand	0.144	0.115
	Jytland (South)	0.208	0.196
	Jytland (Middle)	0.227	0.251
	Jytland (North)	0.095	0.108

Population data from Statistics Denmark
