# Supplementary material for: Citizens’ economic recovery models for a pandemic
Source: PLoS One. 2023 Feb 3;18(2):e0266531. doi: 10.1371/journal.pone.0266531 (PMC9897534; doi:10.1371/journal.pone.0266531)
Supplement: S2 Table — (PDF) [file pone.0266531.s002.pdf]

Economic initiative	Date of adoption or proposal	Description	Budgeted cost
Relief package I	March 10, 2020	Postponement of tax and VAT payments for companies	
Relief package I	March 10, 2020	Compensation for costs relating to the cancellation of larger entertainment events	DKK 2.4 billion
Relief package II	March 12, 2020	Reimbursement of sickness benefits for companies with employees in carantine	
Relief package II	March 12, 2020	Reduced loan restrictions for banks and mortgage institutions	
Relief package II	March 12, 2020	State guarantee on corporate loans	DKK 48.5 billion
Policy proposal	March 13, 2020	Nye Borgerlige proposes payout of 'frozen holiday money'	
Tripartite agreement	March 15, 2020 (extended April 18, 2020 and June 6, 2020)	State salary compensation for private companies	DKK 10.2 billion
Relief package III	March 19, 2020	State compensation for companies' fixed expenses	DKK 65.3 billion
Relief package III	March 19, 2020	State compensation for freelancers and self-employed	DKK 14.1 billion
Relief package III	March 19, 2020	Greater access to loans for students	DKK 0.6 billion
Relief package III	March 19, 2020	Greater access to sickness and unemployment benefits	
Relief package III	March 19, 2020	State guarantee for The Danish Travel Guarantee fund	DKK 1.5 billion
Public investments I	March 26, 2020	Increased budget and investment opportunities for municipalities and regions	
Policy adjustment	March 30, 2020	State salary compensation now cover up to DKK 30.000	
Policy initiative	April 1, 2020	Relief package for universities, evening schools and other educational institutions	DKK 0.17 billion
Policy adjustment	April 18, 2020	Enterprises receiving compensation must not distribute dividend or buy back shares in 2020 and 2021.	
Policy initiative	May 5, 2020	Early payout of tax credit and extended time for payment of tax, payroll tax and VAT	DKK 166.3 billion
Policy initiative	May 5, 2020	Rent-free loans for small and medium-sized companies	DKK 35.4 billion
Policy proposal	May 14, 2020	Venstre demands tax freeze and proposes bringing planned tax cuts of DKK 4.5 billion forward and halving the value-added tax in the rest of 2020	
Public investments II	May 19, 2020	Broad political agreement to allocate DKK 30 billion to green renovations in the social housing sector	DKK 30 billion
Recovery package	June 14, 2020	The creation of a state fund to recapitalize Danish companies and an export package of DKK 500 million	DKK 10.5 billion
Recovery package	June 14, 2020	Payout of 3 weeks of the frozen holiday money and a one-time tax-free supplement of DKK 1.000 to beneficiaries	DKK 27-32 billion
Summer package	June 19, 2020	Discount on tickets and support for cultural institutions and association activities	DKK 0.7 billion
