# Supplementary material for: Citizens’ economic recovery models for a pandemic
Source: PLoS One. 2023 Feb 3;18(2):e0266531. doi: 10.1371/journal.pone.0266531 (PMC9897534; doi:10.1371/journal.pone.0266531)
Supplement: S3 Table — (PDF) [file pone.0266531.s003.pdf]

[illegible]

	Category	#words in dictionary	Share of replies (W1)	Share of replies (W2)
1	demand econ	153	0.233	0.345
2	classical econ	146	0.207	0.057
3	health	129	0.142	0.047
4	dk econ	59	0.068	0.050
5	protect econ	54	0.095	0.100
6	interestrate	53	0.008	0.007
7	helppackages	51	0.212	0.243
8	international econ	47	0.072	0.068
9	climate econ	40	0.021	0.051
10	tax down	39	0.026	0.112
11	social	36	0.037	0.018
12	antipathy	33	0.010	0.011
13	publicspending	28	0.027	0.034
14	non avoidable	20	0.045	0.003
15	austerity	19	0.002	0.003
16	tax up	13	0.017	0.048
17	cashtransfer	12	0.015	0.062
18	bonds	8	0.002	0.002
