# Supplementary material for: Citizens’ economic recovery models for a pandemic
Source: PLoS One. 2023 Feb 3;18(2):e0266531. doi: 10.1371/journal.pone.0266531 (PMC9897534; doi:10.1371/journal.pone.0266531)
Supplement: S4 Table — (PDF) [file pone.0266531.s004.pdf]

	Matches pct. (w1)	Matches pct. (w2)	Matches pct. (w1)	Matches pct. (w2)
	(1)	(2)	(3)	(4)
#words in dictionary	0.0001***	0.0001**	−0.00004	−0.00003
	(0.00004)	(0.0001)	(0.00004)	(0.00005)
Constant	0.022	0.025	0.057**	0.065**
	(0.021)	(0.026)	(0.021)	(0.024)
Std.dev., y	0.077	0.089	0.054	0.063
Excl. top-3 categories	No	No	Yes	Yes
N	18	18	15	15

*p < .1; **p < .05; ***p < .01

Top-3 categories include demand econ, classical econ, and health.
