# Supplementary material for: Citizens’ economic recovery models for a pandemic
Source: PLoS One. 2023 Feb 3;18(2):e0266531. doi: 10.1371/journal.pone.0266531 (PMC9897534; doi:10.1371/journal.pone.0266531)
Supplement: S5 Table — Dictionary measure is a dummy taking the value 1 if a word indicating intervention is identified in the text. Wordscore is a supervised scaling algorithm [22]) that scores the individual answer by comparing it to a set of reference texts, which we code manually. Specifically, we manually code 100 answers with respect to intervention (1) or non-intervention (0). These texts act as anchor for the algorithm to score the remaining texts on a scale ranging from non-intervention to intervention. The association is estimated for four samples: the full sample and three samples excluding observations with the most extreme values on the wordscore measure. This is to prove that the association is not simply driven by clear and obvious (extreme) examples. Interpreting the table, we find a positive and statistically significant association between our dictionary and the wordscore measure. In other words, the two measures of economic intervention are in agreement. The association is furthermore practically important. Moving one standard deviation toward intervention on the wordscore measure corresponds to our dictionary measure being 3pct. to 13pct more likely to identify intervention in the same replies. (PDF) [file pone.0266531.s005.pdf]

	Dictionary measure			
	Full sample	Excl. top/bottom 1 pct.	Excl. top/bottom 3 pct.	Excl. top/bottom 10 pct.
Wordscore	0.096***	0.104***	0.115***	0.137***
	(0.005)	(0.005)	(0.005)	(0.006)
Std. Dev.	3.15	1.86	1.8	1.7
N	13,674	13,440	13,151	12,150

*p < .1; **p < .05; ***p < .01

Dictionary measure is a dummy taking the value 1 if a word indicating intervention is identified in the text. Wordscore is a supervised scaling algorithm (Laver et al. 2003) which scores the individual answer by comparing it to a set of reference texts which we code manually. Specifically, we manually code 100 answers with respect to intervention (1) or non-intervention (0). These texts act as anchor for the algorithm to score the remaining texts on a scale ranging from non-intervention to intervention. The association is estimated for four samples: the full sample and three samples excluding observations with the most extreme values on the wordscore measure. This is to support that the association is not simply driven by clear and obvious (extreme) examples. Interpreting Table 5 Table, we find a positive and statistically significant association between our dictionary and the wordscore measure. In other words, the two measures of economic intervention are in agreement. The association is furthermore practically important. Moving one standard deviation towards intervention on the wordscore measure corresponds to our dictionary measure being 3pct. to 13pct more likely to identify intervention in the same replies.
