# Supplementary material for: Citizens’ economic recovery models for a pandemic
Source: PLoS One. 2023 Feb 3;18(2):e0266531. doi: 10.1371/journal.pone.0266531 (PMC9897534; doi:10.1371/journal.pone.0266531)
Supplement: S6 Table — Ideological right includes Danish Peoples’ Party, Liberals, The Conservative Peoples’ Party, The New Right, and Liberal Alliance. Ideological left is the remaining parties from Fig 4. Unemployed takes the form of a dummy. Education is a numeric variable indicating the highest education obtained (0 = primary school, 8 = PhD). (PDF) [file pone.0266531.s006.pdf]

	Support for economic intervention to combat the Covid19 pandemic					
	Wave 1	Wave 2	Wave 1	Wave 2	Wave 1	Wave 2
	(1)	(2)	(3)	(4)	(5)	(6)
Ideological right	−0.10***	−0.03***	−0.09***	−0.03***	−0.09***	−0.03***
	(0.01)	(0.01)	(0.01)	(0.01)	(0.01)	(0.01)
Age			−0.003***	−0.001***	−0.003***	−0.001***
			(0.0005)	(0.0003)	(0.001)	(0.0003)
Male			−0.01	−0.01	−0.01	−0.01
			(0.01)	(0.01)	(0.01)	(0.01)
Unemployed			−0.01	−0.01	−0.01	−0.01
			(0.02)	(0.01)	(0.02)	(0.01)
Education			0.01**	0.002	0.01**	0.003
			(0.004)	(0.002)	(0.004)	(0.002)
Constant	0.67***	0.91***				
	(0.01)	(0.005)				
Fixed effects	-	-	municipality	municipality	zip code	zip code
N	6,775	6,899	6,557	6,681	6,557	6,681

*p < .1; **p < .05; ***p < .01

Ideological right includes Danish Folkparty, Liberals, The Conservative Folkparty, The New Right, and Liberal Alliance. Ideological left is the remaining parties from Figure **??**. Unemployed takes the form of a dummy. Education is a numeric variable indicating the highest education obtained (0 = primary school, 8 = PhD)
