# Supplementary figures and images for: Citizens’ economic recovery models for a pandemic
Source: PLoS One. 2023 Feb 3;18(2):e0266531. doi: 10.1371/journal.pone.0266531 (PMC9897534; doi:10.1371/journal.pone.0266531)

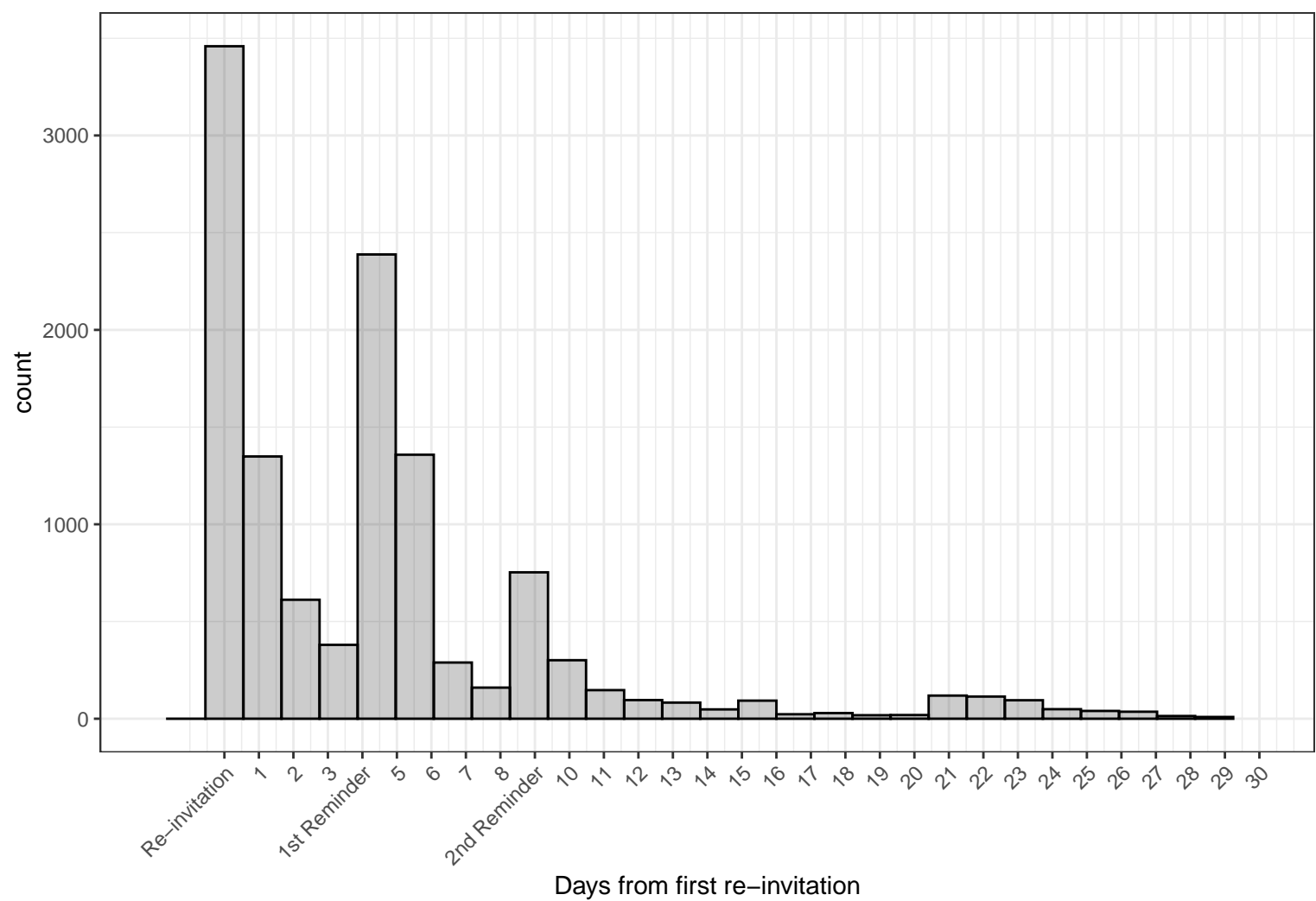

Supplement: S1 Fig — (PDF) [file pone.0266531.s007.pdf]

Wave one Wave two

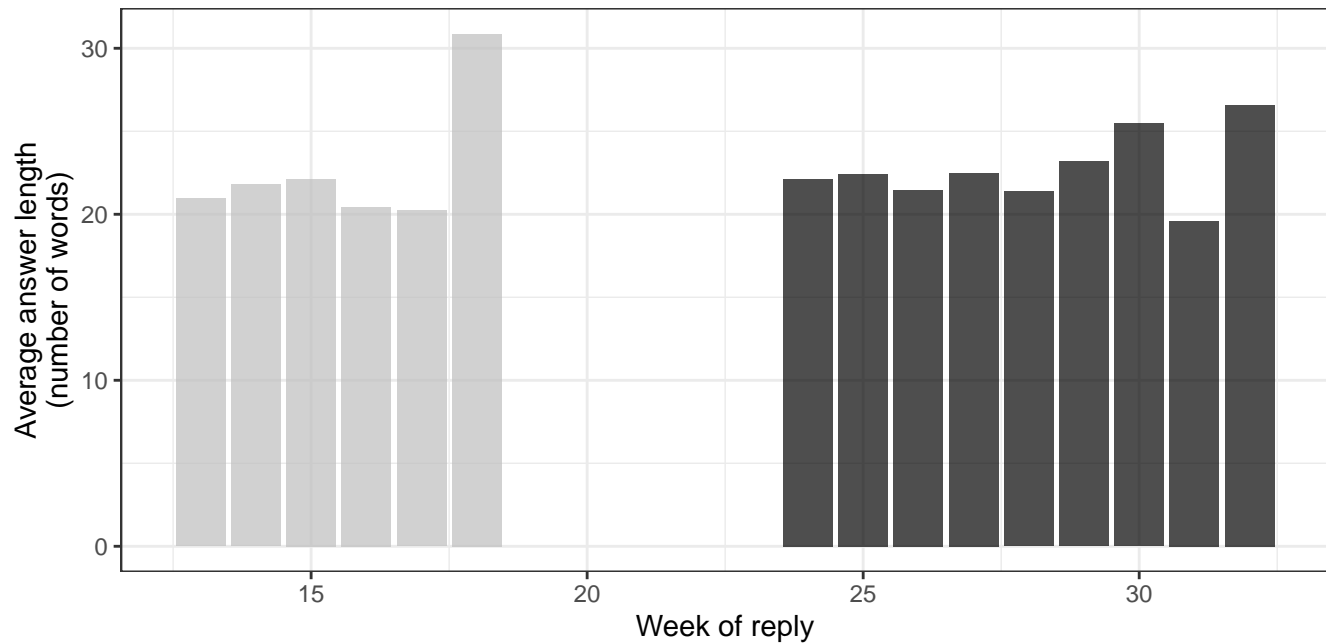

Supplement: S3 Fig — (PDF) [file pone.0266531.s009.pdf]

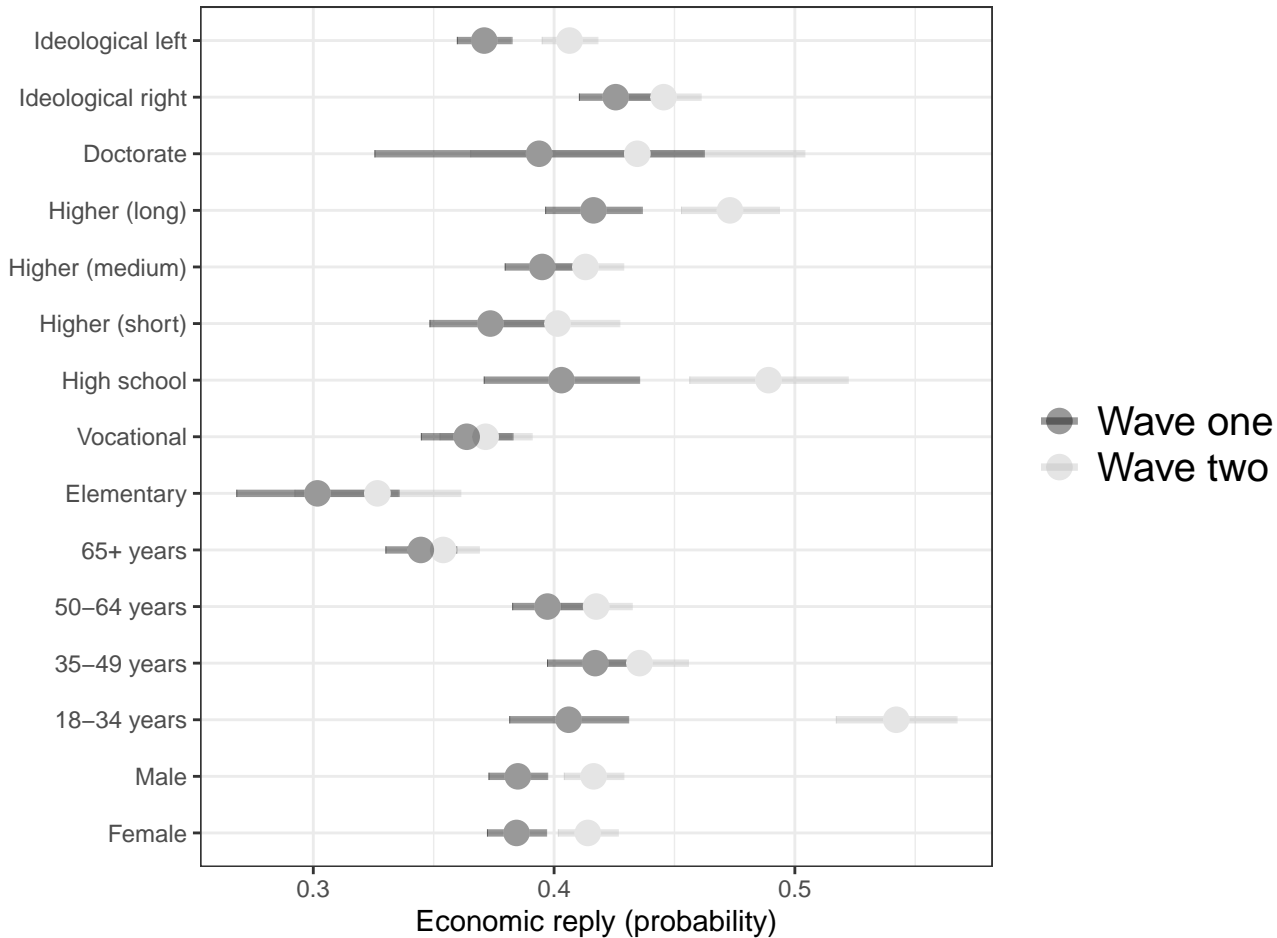

Supplement: S4 Fig — (PDF) [file pone.0266531.s010.pdf]

Within-respondent difference  
between wave one and wave two

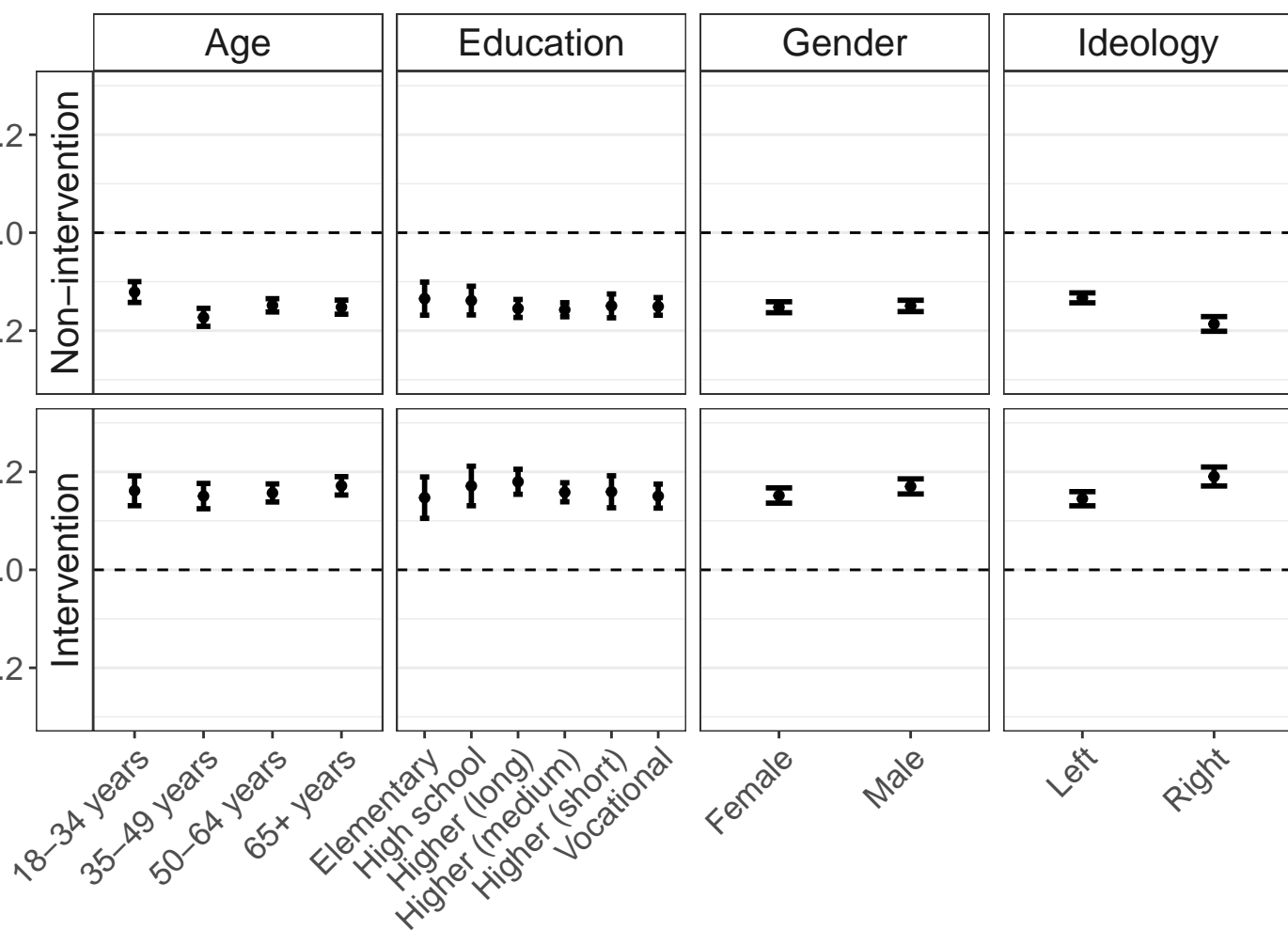

Supplement: S5 Fig — (PDF) [file pone.0266531.s011.pdf]

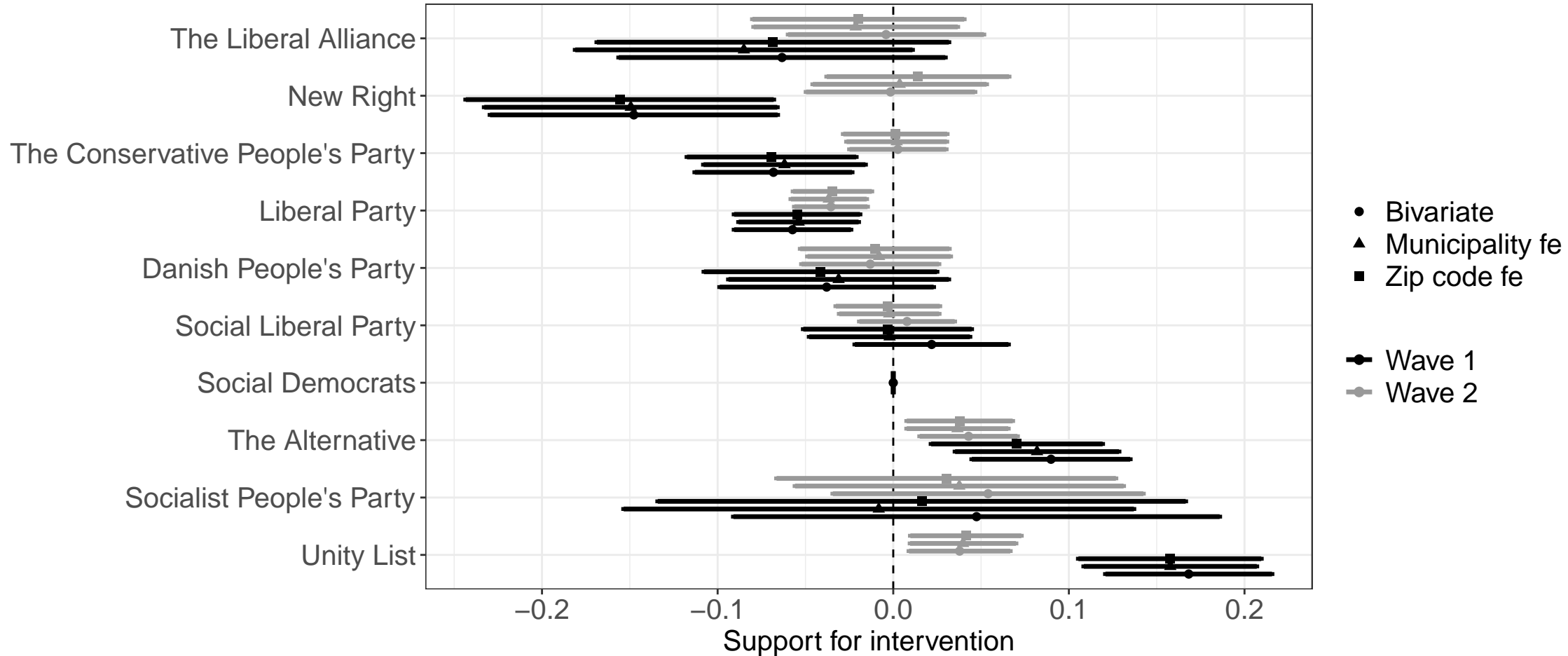

Supplement: S6 Fig — Party positions relative to the Social Democrats (governing party). Models control for age, gender, employment status, and education and include fixed effects. (PDF) [file pone.0266531.s012.pdf]
