# Supplementary material for: Citizens’ economic recovery models for a pandemic
Source: PLoS One. 2023 Feb 3;18(2):e0266531. doi: 10.1371/journal.pone.0266531 (PMC9897534; doi:10.1371/journal.pone.0266531)
Supplement: S2 Fig — (PDF) [file pone.0266531.s008.pdf]

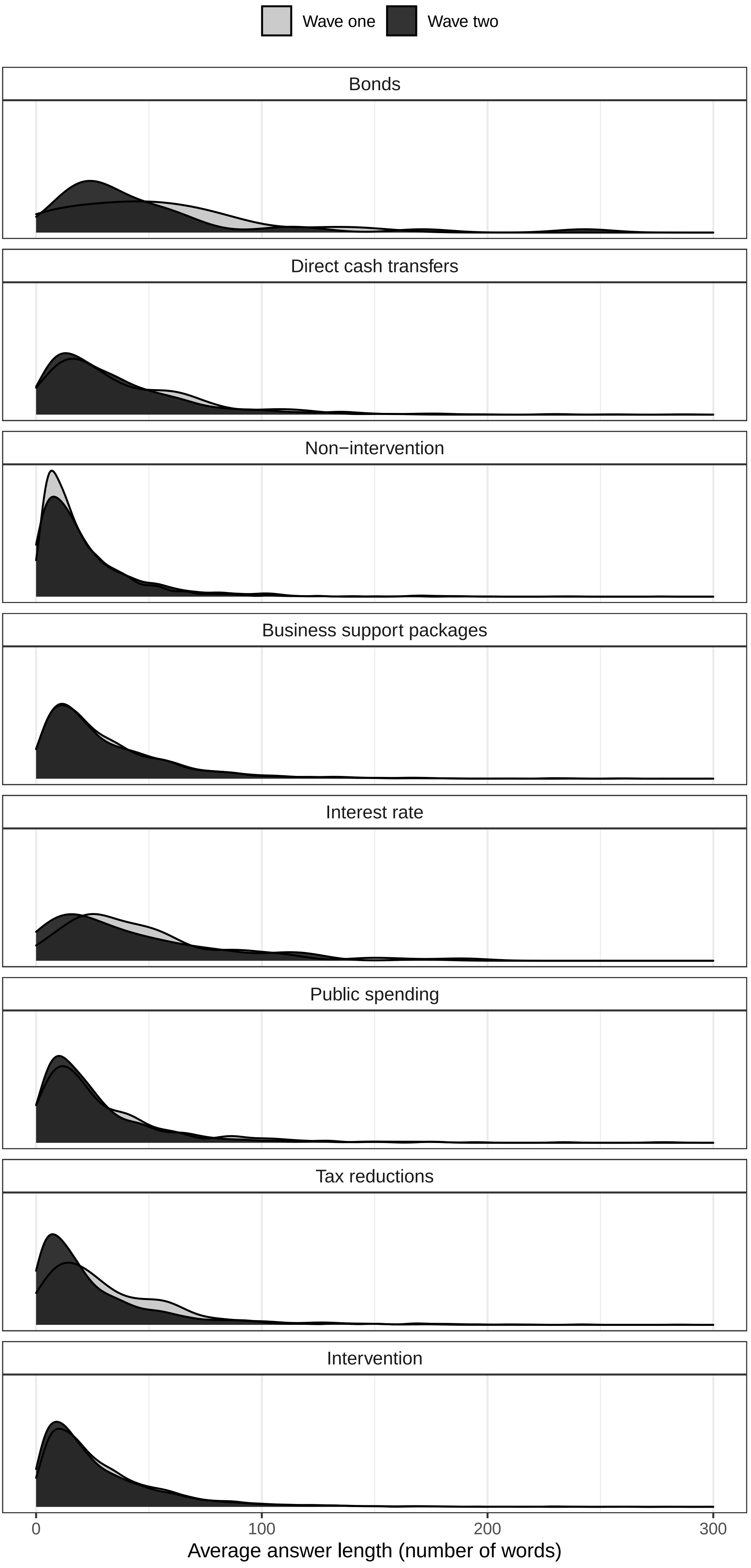

Wave one
Wave two
Bonds
Direct cash transfers
Non−intervention
Business support packages
Interest rate
Public spending
Tax reductions
Intervention
0
100
200
300
Average answer length (number of words)
